# Warthin-Like Papillary Thyroid Carcinoma Associated with Lymphadenopathy and Hashimoto's Thyroiditis

**DOI:** 10.1155/2015/251898

**Published:** 2015-03-03

**Authors:** Karla Judith González-Colunga, Abelardo Loya-Solis, Luis Ángel Ceceñas-Falcón, Oralia Barboza-Quintana, René Rodríguez-Gutiérrez

**Affiliations:** ^1^Pathology Department, University Hospital “Dr. José E. González” and Medical School of the Autonomous University of Nuevo Leon, Madero and Gonzalitos s/n, 64460 Monterrey, Nuevo León, Mexico; ^2^Endocrinology Division, University Hospital “Dr. José E. González” and Medical School of the Autonomous University of Nuevo Leon, Madero and Gonzalitos s/n, 64460 Monterrey, Nuevo León, Mexico

## Abstract

Defining the histologic variant of thyroid carcinoma is an important clinical implication as their progression, recurrence, aggressiveness, and prognosis differ. Warthin-like variant is one of the rarest histologic variants of papillary thyroid cancer. A 36-year-old female sought consult for assessment of a painless right neck tumor. High-resolution neck ultrasound revealed a right hypoechoic, 1.71 × 1.05 cm thyroid nodule. Ultrasound-guided fine-needle aspiration biopsy report was a Bethesda grade III. Thyroid function tests showed Hashimoto's thyroiditis. The patient underwent right hemithyroidectomy. Microscopically, the tumor was composed of papillae lined by cells with eosinophilic cytoplasm, nuclear chromatin clearing, grooves, and pseudoinclusions and a characteristic lymphoplasmacytic infiltrate of the papillae cores. Extension into the perithyroidal soft tissue and 3 ipsilateral lymph nodes was found to be positive for cancer. Warthin-like variant is an uncommon and relatively unknown variant of papillary thyroid carcinoma that has been usually associated with an excellent prognosis. Interestingly, BRAF mutations have been reported to be present in up to 75% of the patients. It is frequently associated with Hashimoto's thyroiditis and presents unique morphological features that make it recognizable on histologic examination. The cytological diagnosis is difficult to assess due to the overlap in its findings with the classical variant and Hashimoto's thyroiditis.

## 1. Introduction

Papillary, follicular, and anaplastic thyroid cancers are follicular epithelial-derived cancers. Papillary and follicular cancers are considered differentiated cancers and patients with these tumors are treated similarly, nevertheless being biologically different [1, 2]. On their pathogenesis, proteins in the mitogen-activated protein kinase (MAPK) pathway have gained interest as almost 70% of differentiated thyroid cancers may present exclusive nonoverlapping activation mutations in BRAF, RET, or RAS [3–6]. Several histologic subtypes of papillary thyroid cancer (PTC) have been described. Of these the follicular variant is the most common and the so-called “Warthin-like” (WL) variant has been seldom reported [7, 8].

Defining the histologic variants of thyroid carcinoma is an important clinical implication as their progression, recurrence, aggressiveness, and prognosis differ [9–12]. Nevertheless, in many centers around the world the possibility of such histologic resolution is not available. Moreover, scarce information can be found in the literature; consequently their behaviors are not completely understood and specific target goals in their evaluation and treatment are lacking. Warthin-like variant is one of the rarest histologic variants of papillary thyroid cancer that has been seldom reported and that has been classically described in females over 50 years with a similar clinical presentation and prognosis as the classic papillary thyroid cancer [7, 8].

Herein we present the case of a 36-year-old female with a Warthin-like papillary thyroid carcinoma with positive lymphadenopathy for cancer.

## 2. Case Report

A 36-year-old female sought consult for assessment and management of a one-year history of an increasing painless right neck tumor. She had a past medical history of gestational diabetes mellitus during her first delivery and was otherwise healthy. Also she denied having a family history of thyroid disease, exposure to irradiation, or any other risk factor associated with thyroid cancer.

**Figure 1:**
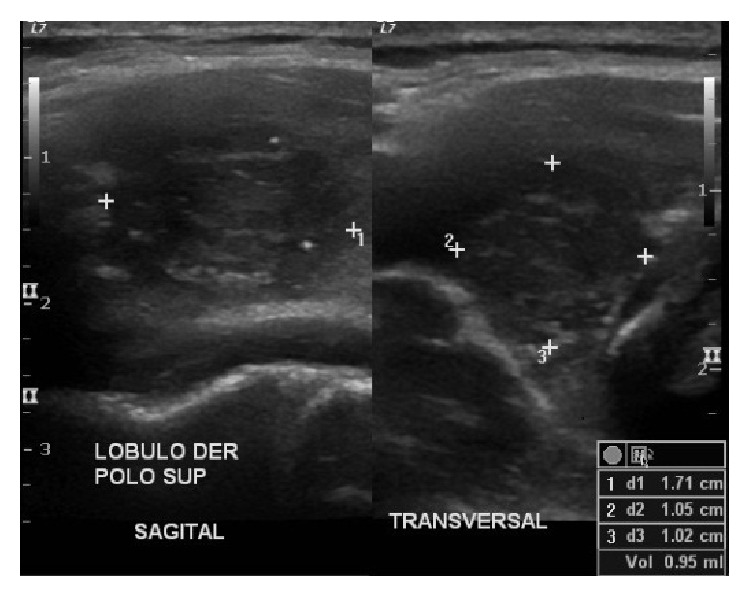
Neck ultrasound showing a hypoechoic nodule in the inferior pole of the right thyroid lobe, measuring 1.71 × 1.05 cm, characterized by irregular margins and accompanied by microcalcifications.

**Figure 2:**
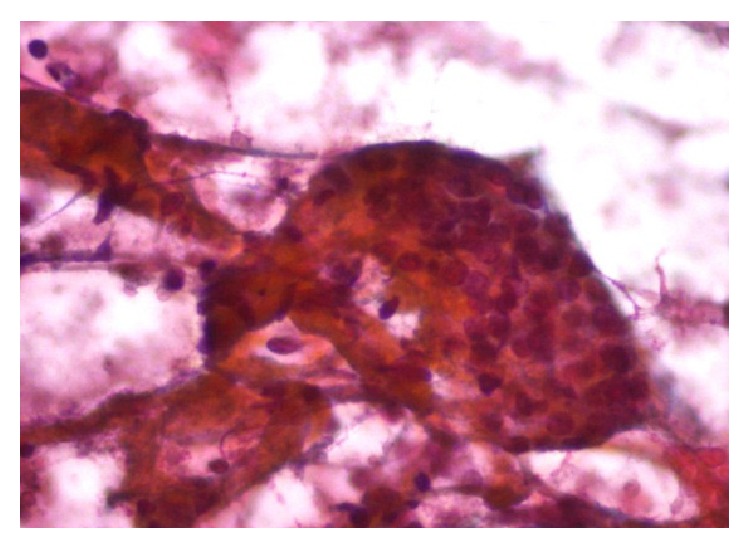
Irregular cell cluster with atypical cytological features (Papanicolaou stain, 200x).

**Figure 3:**
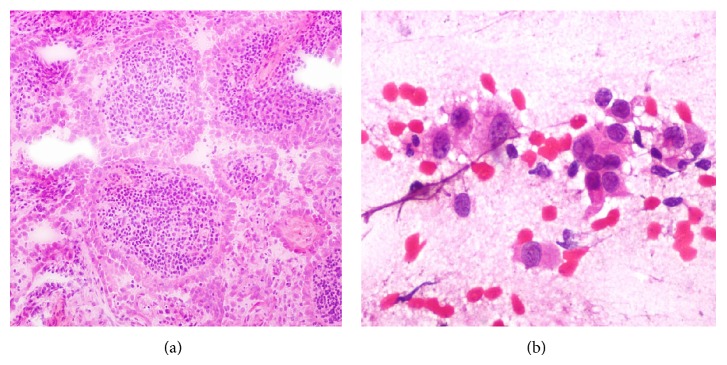
(a) Transverse section of papillae lined by an oncocytic epithelium and characteristic lymphoplasmacytic infiltrate of the papillae cores (frozen section and hematoxylin and eosin stain, 40x). (b) Intraoperative smear cytology showing cells with eosinophilic cytoplasm and nuclear pseudoinclusions (hematoxylin and eosin stain, 400x).

**Figure 4:**
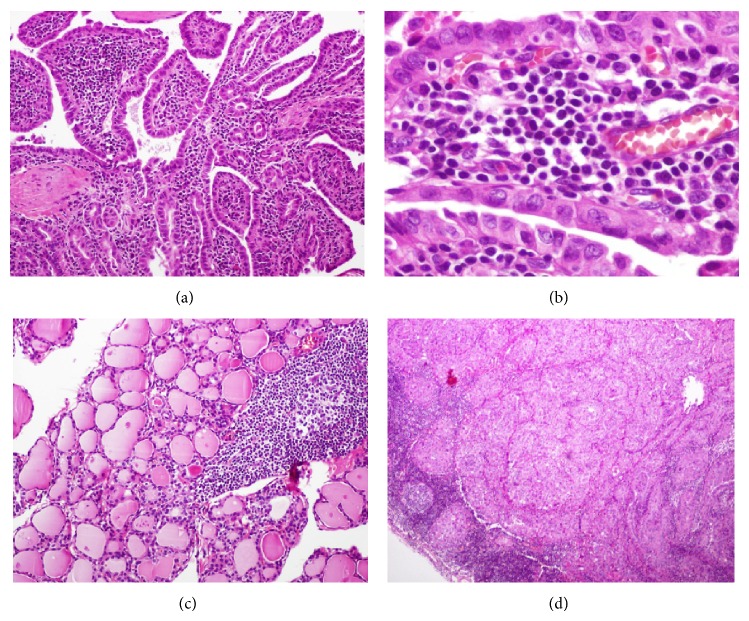
(a) Papillae lined by cells with eosinophilic cytoplasm and a lymphoplasmacytic infiltrate of the cores (hematoxylin and eosin stain, 40x). (b) Nuclear features of papillary carcinoma (nuclear grooves) (hematoxylin and eosin stain, 200x). (c) Lymphocytic thyroiditis in nonneoplastic areas of thyroid (hematoxylin and eosin stain, 40x). (d) Lymph node metastasis (hematoxylin and eosin stain, 40x).

Physical examination revealed painless, nonfixed, regular, and hard nodule of 1 × 2 cm in the inferior right thyroid lobe (zone VI). Dyspnea, dysphagia, and dysphonia were denied and cervical lymphadenopathy was absent. High-resolution neck ultrasound revealed a right hypoechoic, 1.71 × 1.05 cm thyroid nodule with irregular margins, central vascularity, incomplete halo, and microcalcifications (Figure 1). Ultrasound-guided fine-needle aspiration biopsy was performed and the cytology report was a Bethesda grade III (atypia of undetermined significance) (Figure 2) [2]. Thyroid function tests showed subclinical hypothyroidism, TSH = 5.19 mlUI/ml (0.27–4.2 mlUI/ml), free T4 of 1.24 ng/dl (0.93–1.7 ng/dl), normal total T3 and T4, and positive thyroperoxidase antibodies (Hashimoto's thyroiditis). Full thyroid hormone replacement therapy was initiated and surgery was planned. The patient underwent right hemithyroidectomy with intraoperative cytology and frozen section evaluation, which reported a papillary carcinoma (Figure 3). In view of the intraoperative diagnosis, a total thyroidectomy was performed. Gross pathological analysis of the thyroid showed solid, infiltrative, and ill-defined white tumor of 1.7 cm in its greatest diameter localized in the inferior pole of the right thyroid lobe. Microscopically the tumor was composed of papillae lined by cells with eosinophilic cytoplasm, nuclear chromatin clearing, grooves, and pseudoinclusions and a characteristic lymphoplasmacytic infiltrate of the papillae cores (Figure 4). Extension into the perithyroidal soft tissue and 3 ipsilateral lymph nodes was found to be positive for cancer. Based on these findings the diagnosis was Warthin-like variant of a papillary thyroid carcinoma. There were no postoperative complications and the patient was discharged three days after admission. Two months later she underwent I-131 radioablative therapy (100 mCi) with a 7-day posttreatment tracing that showed scarce uptake of radioiodine in the thyroid area. At 12-month follow-up the patient was asymptomatic, neck ultrasound was negative for recurrences, and stimulated thyroglobulin was within expected goals.

## 3. Discussion

World Health Organization (WHO) recognizes 9 main histopathological papillary thyroid cancer variants: follicular, macrofollicular, oncocytic, clear cell, diffuse sclerosing, tall cell variant, columnar cell, solid, and cribriform [13]. The most recent edition of the WHO classification of tumors of endocrine organs classifies “Warthin-like tumor” under the oncocytic variant section. We believe it is important to acknowledge all these variants due to a more aggressive biological behavior of at least two of them (tall and columnar cell variants) [14, 15]. The Warthin-like variant is morphologically characterized by a papillary architecture with an oncocytic epithelial lining and a lymphoplasmacytic core infiltrate [16]. Also important to mention is its well-known association to Hashimoto's thyroiditis present in the nonneoplastic thyroid tissue [13, 17]. Both the oncocytic and tall cell variants share a morphological papillary architecture, having two main differences: the lack of lymphoplasmacytic infiltrate on the former and more elongated oncocytes with a height that is more than three times their width in the latter [13, 16, 18]. Vera-Sempere et al. proposed that the Warthin-like variant is a hybrid of the tall cell and oncocytic variants [19].

A cytological diagnosis is possible and has been reported in the literature; however since its findings are those of the classic variant of the papillary carcinoma (papillary clusters, monolayered sheets of oncocytic cells with ground glass appearance, nuclear grooves, and intranuclear pseudoinclusions) and Hashimoto's thyroiditis (lymphocyte rich background), the correct diagnosis can be really difficult for a pathologist if there is no single dominant feature of such findings [20].

Regarding Warthin-like variant age distribution, it has been usually described that its presentation is a decade earlier than the classic variant, however other case series have reported the opposite [17, 21]. When compared with the classic variant a significant difference in prognosis has not been proven, since Warthin-like prognosis seems to be also excellent [21]. The presence of regional lymph node metastases is uncommon in Warthin-like variant and only 3 out of the 13 patients in the original case series reported by Apel presented with it [7]. In larger case series, lymphadenopathy has been reported in up to 22% of the cases [21]. At the time of diagnosis our patient had positive right regional lymph node, however she had an excellent response to the treatment and at 12-month follow-up she remains clinically, radiologically, and biochemically free of disease.

BRAF mutations (substitution of a valine for a glutamic acid (V599E)) have been implicated in the pathogenesis of papillary thyroid cancer and had been reported to be present in up to 50% of the cases [22]. They usually confer worst clinical prognosis as they are associated with a more extensive disease and a higher recurrence rate. In this sense, it is interesting that BRAF mutations have been reported to be present in up to 75% of the patients with Warthin-like variant that as we have previously mentioned have been usually known to have an excellent prognosis [23]. Moreover, our patient presented with lymphadenopathy that could have important clinical implications in her prognosis in the future. This highlights the necessity of having the histologic subtypes in all cases as many cases that might be considered to be aggressive “classic variants” might as well be Warthin-like variants or other known aggressive subtypes of papillary thyroid cancer.

## 4. Conclusion

Warthin-like variant is an uncommon and relatively unknown variant of papillary thyroid carcinoma. It is frequently associated with Hashimoto's thyroiditis and presents unique morphological features that make it easily recognizable on the histologic examination. The cytological diagnosis is difficult to assess due to the overlap in its findings with the classical variant and Hashimoto's thyroiditis. The scarce case series have suggested that age group, gender distribution, and prognosis are the same as those reported for the classic variant. However BRAF mutations have been reported to be present in up to 75% of the patients with Warthin-like variant. Consequently, further studies with larger series and long-term monitoring are required to establish this with certainty.
